# Seed Priming with Chitosan Improves Germination Characteristics Associated with Alterations in Antioxidant Defense and Dehydration-Responsive Pathway in White Clover under Water Stress

**DOI:** 10.3390/plants11152015

**Published:** 2022-08-02

**Authors:** Yao Ling, Yue Zhao, Bizhen Cheng, Meng Tan, Yan Zhang, Zhou Li

**Affiliations:** Department of Turf Science and Engineering, College of Grassland Science and Technology, Sichuan Agricultural University, Chengdu 611130, China; ly9729752@163.com (Y.L.); zhaoyue19971120@126.com (Y.Z.); chengbizhengrass@163.com (B.C.); tanmeng194@163.com (M.T.); zhangyan1111zy@126.com (Y.Z.)

**Keywords:** antioxidant enzyme, dehydrins, *DREB* transcription factor, oxidative damage, seed vigor, reactive oxygen species

## Abstract

Water stress decreases seed-germination characteristics and also hinders subsequent seedling establishment. Seed priming with bioactive compounds has been proven as an effective way to improve seed germination under normal and stressful conditions. However, effect and mechanism of seed priming with chitosan (CTS) on improving seed germination and seedling establishment were not well-understood under water-deficit conditions. White clover (*Trifolium repens*) seeds were pretreated with or without 5 mg/L CTS before being subjected to water stress induced by 18% (*w*/*v*) polyethylene glycol 6000 for 7 days of germination in a controlled growth chamber. Results showed that water stress significantly decreased germination percentage, germination vigor, germination index, seed vigor index, and seedling dry weight and also increased mean germination time and accumulation of reactive oxygen species, leading to membrane lipid peroxidation during seed germination. These symptoms could be significantly alleviated by the CTS priming through activating superoxide dismutase, catalase, and peroxidase activities. In addition, seeds pretreated with CTS exhibited significantly higher expression levels of genes encoding dehydration-responsive transcription factors (*DREB2*, *DREB4*, and *DREB5*) and dehydrins (*Y2K*, *Y2SK*, and *SK2*) than those seeds without the CTS priming. Current findings indicated that the CTS-induced tolerance to water stress could be associated with the enhancement in dehydration-responsive pathway during seed germination.

## 1. Introduction

With the development of global warming, drought stress has become one of the destructive environmental factors affecting seed germination and plant growth worldwide [1]. Seed germination and seedling establishment are key stages of plant growth and development but are also very vulnerable to drought stress [2]. Drought reduced seed-germination rate and subsequent seedlings establishment, leading to yield loss and quality deterioration [3]. Cell dehydration is one of main adverse effects induced by drought. Alteration of dehydration-responsive pathway is a universal response to water deficit in the plant kingdom [4]. For example, dehydration-responsive element-binding proteins (DREBs) recognize and bind to the dehydration-responsive element (DRE) of many downstream stress-responsive genes such as dehydrins (DHNs), which is an important adaptive strategy when plants survive under drought stress [5]. It has been widely reported that the overexpression of *DREBs* up-regulated transcriptional levels of different types of *DHNs*, thereby enhancing drought tolerance in many plants such as *Arabidopsis thaliana*, rice (*Oryza sativa* L.), tobacco (*Nicotiana tabacum* L.), and wheat (*Triticum aestivum* L.) [6,7]. Wheat seed pretreated with microbe *Bacillus* sp. or *Klebsiella* sp. effectively mitigated drought-induced declines in seedling biomass and root growth associated with significant up-regulation of *DHN* and *DREB* [8].

Oxidative damage induced by overaccumulation of reactive oxygen species (ROS) such as superoxide anion (O_2_^−^) and hydrogen peroxide (H_2_O_2_) is another serious consequence when seeds germinate and seedlings establish under drought condition [9]. Rapid detoxification of O_2_^−^ and H_2_O_2_ by regulating antioxidant defense has been recognized as one of pivotal adaptive mechanisms of drought tolerance in plants. As key components of antioxidant defense, superoxide dismutase (SOD) catalyzes dismutation of O_2_^−^ into H_2_O_2_ and O_2_, and catalase (CAT), peroxidase (POD), and ascorbate peroxidase (APX) reduce H_2_O_2_ to nontoxic H_2_O [10]. It has been found that drought-tolerant alfalfa (*Medicago sativa* L.) cultivar Xinmu No.1 accumulated lower H_2_O_2_ and malondialdehyde (MDA) contents through activating SOD, CAT, POD, and APX activities during seed germination under drought stress [11]. In response to drought, better antioxidant capacity and less ROS accumulation in soybean (*Glycine max* L.) seedlings were positively correlated with higher seed-germination rate [12]. Exogenous silicon could improve tomato (*Lycopersicon esculentum* L.) seed germination in relation to enhanced antioxidant enzymes activities and reduced oxidative stress [13]. These findings indicated the importance of effective antioxidant defense during seed germination and seedling establishment under water deficit condition.

Chitosan (CTS) is a bioactive compound from plants and marine crustaceans, such as crab shells and waste shrimp. In recent years, the CTS has been widely used in agricultural and horticultural fields for the improvement in crop quality and stress adaptation due to its non-toxic and biodegradable property [14]. It has been reported that the CTS could trigger many defensive responses to drought in plants. For example, exogenous application of CTS helped to maintain functional and structural integrity of biological membranes associated with increases in CAT and APX activities and the accumulation of secondary metabolite in periwinkle (*Catharanthus roseus* L.) [15]. Seed soaking with CTS could increase the accumulation of indoleacetic acid and free amino acids in favor of subsequent lupine (*Lupinus termis* L.) growth and yield under drought stress [16]. Foliar application of CTS also could effectively alleviate drought-induced growth inhibition of lettuce (*Lactuca sativa* L.) plants [17]. In addition, the exogenous CTS significantly increased photosynthetic rate, water use efficiency, and CAT, POD, and SOD activities in pot marigold (*Calendula officinalis* L.) plants, thereby mitigating deleterious effect of drought stress on growth [18].

Seed priming with bioactive compounds or elements such as zinc, γ-aminobutyric acid (GABA), putrescine (Put), diethyl aminoethyl hexanoate (DA-6), or spermidine (Spd) has been proven as an effective way to improve seed germination under normal and stressful conditions [19,20,21,22,23]. Previous studies have found that chitosan-black soybean seed-coat extract exhibited strong antioxidant property, and CTS coating could effectively improve seed germination, seedling growth, and resistance to pests under normal condition [24,25,26]. However, research has still not fully elucidated the effect of seed priming with CTS on alleviating drought-induced damage to seed germination and seedling establishment. White clover (*Trifolium repens* L.) is an important forage for feeding livestock and also used as an ornamental plant in horticulture. Objects of this study were to investigate the effect of CTS priming on seed-germination characteristics and to further elucidate the underlying mechanism involved in antioxidant defense and the dehydration-responsive pathway during white clover seed germination under water stress. Current findings will be beneficial to better understand the CTS-regulated adaptability to water stress during seed germination.

## 2. Materials and Methods

### 2.1. Plant Materials and Treatments

Seeds (white clover cv. Haifa) were surface-sterilized in 0.1% HgCl_2_ solution for 5 min and then rinsed four times in distilled water (ddH_2_O). These seeds were divided into two groups: one group was soaked in ddH_2_O for 3 h (seeds without the CTS priming), and another group was firstly soaked in ddH_2_O for 1 h and then transferred into 5 mg/L CTS (Sigma-Aldrich, 900344, St. Louis, MO, USA) solution for 2 h (seeds priming with the CTS). Seeds primed with or without the CTS were then germinated in Petri dishes. Three sheets of filter papers were laid in each Petri dish and moistened with 15 mL of ddH_2_O (normal germination condition) or 18% (*w*/*v*) polyethylene glycol 6000 (PEG 6000) solution (germination under water stress). Each treatment included six biological replications, and each Petri dish included 50 seeds. All Petri dishes were placed randomly in a growth chamber (average day/night temperature of 23/19 °C, 75% relative humidity, and 700 μmol·m^−2^·s^−1^ photosynthetically active radiation (PAR) at 12 h photoperiod) for 7 days. Seedlings were sampled on the 7th day of germination for determination of growth, physiological parameters, and gene expression levels.

### 2.2. Measurements of Seed-Germination and Growth Parameters

Germination vigor (GV) or germination percentage (GP) was calculated as a percentage of those seeds that had germinated on the 3rd or 7th day after the start of H_2_O or CTS pretreatment, respectively. The germination index (GI) was calculated based on the formula:

∑(Gt/Dt)(1)

Gt indicates the number of germinated seeds, and Dt indicates the corresponding time to Gt in days.

Mean germination time (MGT) was calculated based on the formula:

MGT=∑(D × n)/∑n(2)

D indicates the number of days, and N indicates the number of germinations in the corresponding days.

For root length (RL), shoot length (SL), fresh weight (FW), and dry weight (DW), 10 seedlings were randomly selected from each treatment after 7 days of germination. Seed vigor index (SVI) was the product of FW and GI [22].

### 2.3. Measurements of Reactive Oxygen Species and Antioxidant Enzyme Activities

Superoxide anion (O_2_^−^) or hydrogen peroxide (H_2_O_2_) content was determined according to the method of Elstner and Heupel [27] or Velikova et al. [28], respectively. For malondialdehyde (MDA) content and antioxidant enzyme activity, 0.2 g of fresh seedlings were homogenized with 3 mL of 50 mM cold phosphate buffer (pH 7.8) and then centrifuged at 10,000× *g* for 15 min at 4 °C. The supernatant was collected for MDA determination and also as enzyme extract. The MDA content was determined by using 0.5 mL of the supernatant and 1 mL of the reaction solution (20% *w*/*v* trichloroacetic acid and 0.5% *w*/*v* thiobarbituric acid). After being heated in a boiling water for 15 min, the reaction mixture was cooled down to room temperature, and the absorbance of reaction solution was measured at 532 and 600 nm by using a spectrophotometer (Spectronic Instruments, Rochester, NY, USA) [29].

For SOD activity, 0.05 mL of supernatant was mixed with 1.45 mL of 50 mM phosphate solution (pH 7.8) containing 1.125 μM NBT, 60 μM riboflavin, 195 mM methionine, and 3 μM EDTA. After being placed under 600 µmol m^−2^·s^−1^ PAR for 10 min, the absorbance of reaction solution were detected at 560 nm [30]. POD and CAT activities were detected based on the method of Chance and Maehly [31]. Briefly, 0.05 mL of supernatant was mixed with 1 mL of 50 mM phosphate buffer (pH 7.0) containing 45 mM H_2_O_2_ solution, and then, the absorbance of reaction solution was detected at 240 nm for the CAT activity. The 0.025 mL of supernatant was mixed with 0.05 mL of H_2_O_2_ solution, 0.5 mL guaiacol solution, and 0.925 mL phosphate buffer (pH 7.0). The absorbance of reaction solution was detected at 470 nm for the POD activity. For APX activity, 0.05 mL of supernatant was mixed with 100 mM of sodium acetate buffer (pH 5.8), 10 mM ascorbic acid, 5 mM H_2_O_2_, and 0.003 mM ethylenediaminetetraacetic acid, and then, the absorbance of reaction solution was detected at 290 nm [32].

### 2.4. Measurements of Genes Expression Levels

Fresh seedlings (0.15 g) were sampled for total RNA extraction using a total RNA extraction kit (Tiangen, China), and then, the RNA were reverse-transcripted into cDNA (PrimeScript™ RT reagent Kit with gDNA Eraser, TaKaRa, Japan). Primers of *β*-Actin (internal reference gene) and genes encoding different types of dehydrins and dehydration-responsive element-binding proteins (Table 1) were used for real-time quantitative fluorescence PCR (qRT-PCR). The PCR procedure for all genes was: 94 °C for 5 min, denaturation at 95 °C for 30 s (40 repeats), annealing at 58 or 60 °C (Table 1) for 30 s, and extension at 72 °C for 30 s. Genes’ relative expression levels were calculated by using the formula 2^−ΔΔCt^ [33].

### 2.5. Statistical Analysis

Variations among four treatments were analyzed by the general linear model procedure of Statistical Product and Service Solutions 24 (SPSS Institute, IBM, Armonk, NY, USA, 2018). Differences among treatments were determined by using the least significant difference (LSD) at *p* ≤ 0.05.

## 3. Results

### 3.1. Seeds Priming with CTS Affected Germination Characteristics under Water Stress

**Figure 1:**
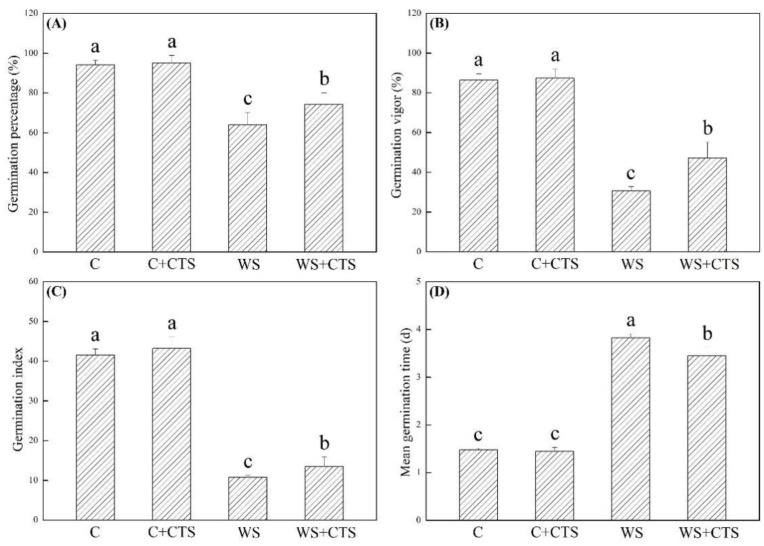
Seeds priming with chitosan affected (**A**) germination percentage, (**B**) germination vigor, (**C**) germination index, and (**D**) mean germination time under water stress. Vertical bars above columns indicate ± SE of mean, and different letters above columns indicate significant difference (*p* < 0.05). C, control (seeds pretreated with H_2_O germinated under normal condition); C+CTS, control + CTS (seeds pretreated with CTS germinated under normal condition); WS, water stress (seeds pretreated with H_2_O germinated under water stress condition); WS+CTS, water stress + CTS (seeds pretreated with CTS germinated under water stress condition).

**Figure 2:**
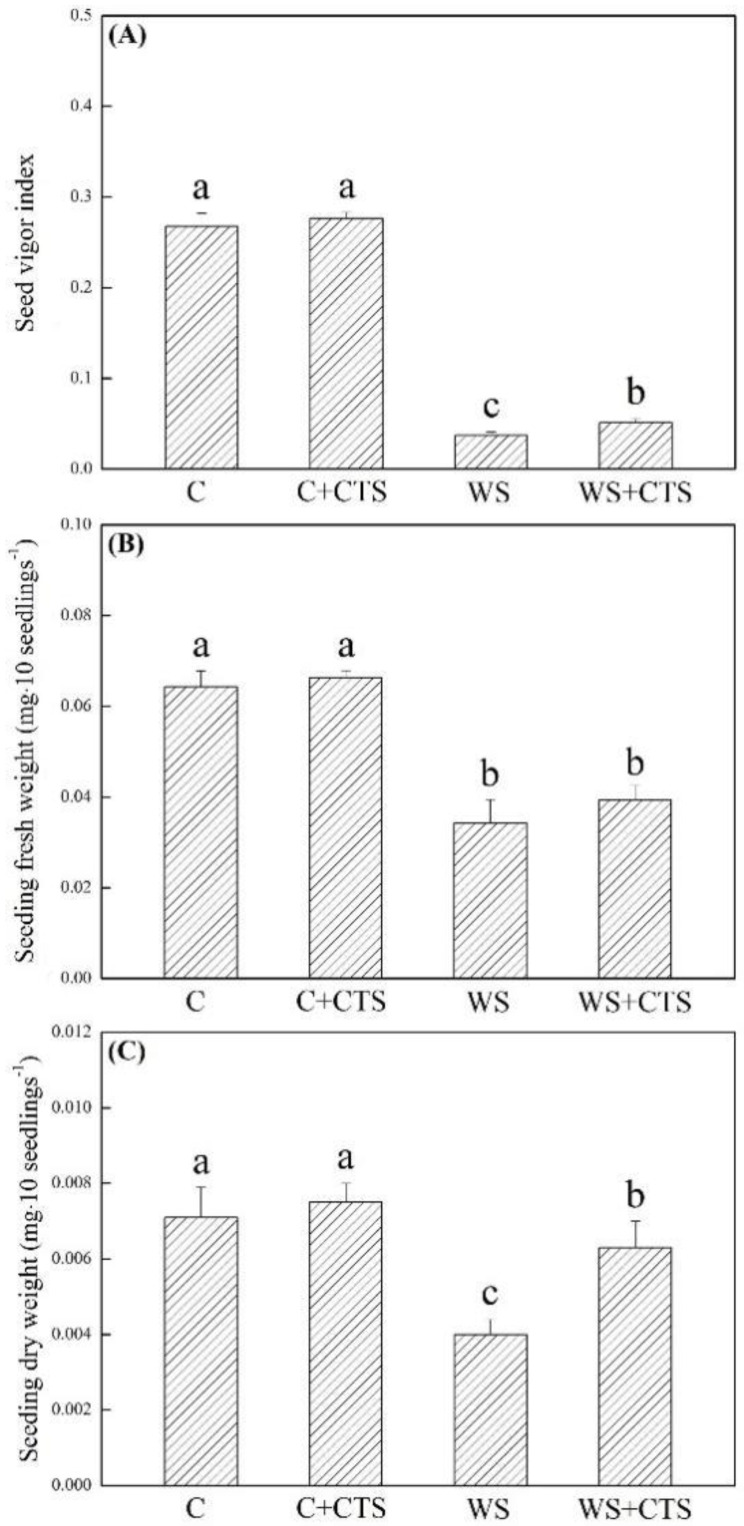
Seed priming with chitosan affected (**A**) seed vigor index, (**B**) seedling fresh weight, and (**C**) seedling dry weight after 7 days of germination under water stress. Vertical bars above columns indicate ± SE of mean, and different letters above columns indicate significant difference (*p* < 0.05). C, control (seeds pretreated with H_2_O germinated under normal condition); C+CTS, control + CTS (seeds pretreated with CTS germinated under normal condition); WS, water stress (seeds pretreated with H_2_O germinated under water stress condition); WS+CTS, water stress + CTS (seeds pretreated with CTS germinated under water stress condition).

**Figure 3:**
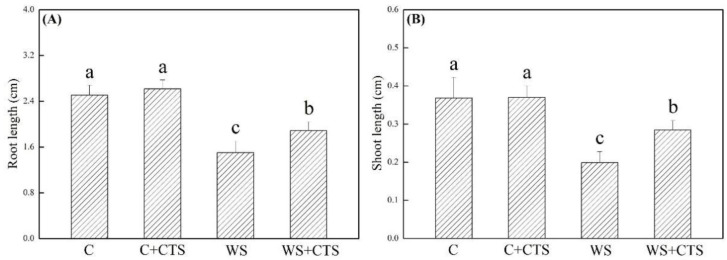
Seed priming with chitosan affected (**A**) seedling root length and (**B**) seedling shoot length after 7 days of germination under water stress. Vertical bars above columns indicate ± SE of mean, and different letters above columns indicate significant difference (*p* < 0.05). C, control (seeds pretreated with H_2_O germinated under normal condition); C+CTS, control + CTS (seeds pretreated with CTS germinated under normal condition); WS, water stress (seeds pretreated with H_2_O germinated under water stress condition); WS+CTS, water stress + CTS (seeds pretreated with CTS germinated under water stress condition).

GP, GV, GI, and MGT were not significantly affected by the CTS priming under normal conditions (Figure 1A–D). PEG-induced water stress resulted in significant declines in GP, GV, GI, and MGT of seeds primed with or without CTS. Seeds primed with CTS exhibited a 16%, 54%, or 26% greater increase in GP, GV, or GI than those seeds without the CTS priming under water stress, respectively (Figure 1A–C). Seeds primed with CTS showed significantly lower MGT than seeds without the CTS priming under water stress (Figure 1D). As compared to normal condition, water stress also significantly decreased SVI, FW, DW, RL, and SL (Figure 2A–C and Figure 3A,B). However, seeds primed with CTS had significantly higher SVI, DW, RL, and SL than those seeds without the CTS priming after 7 days of germination under water stress (Figure 2A,C and Figure 3A,B).

### 3.2. Seed Priming with CTS Affected Oxidative Damage and Antioxidant Defense under Water Stress

**Figure 4:**
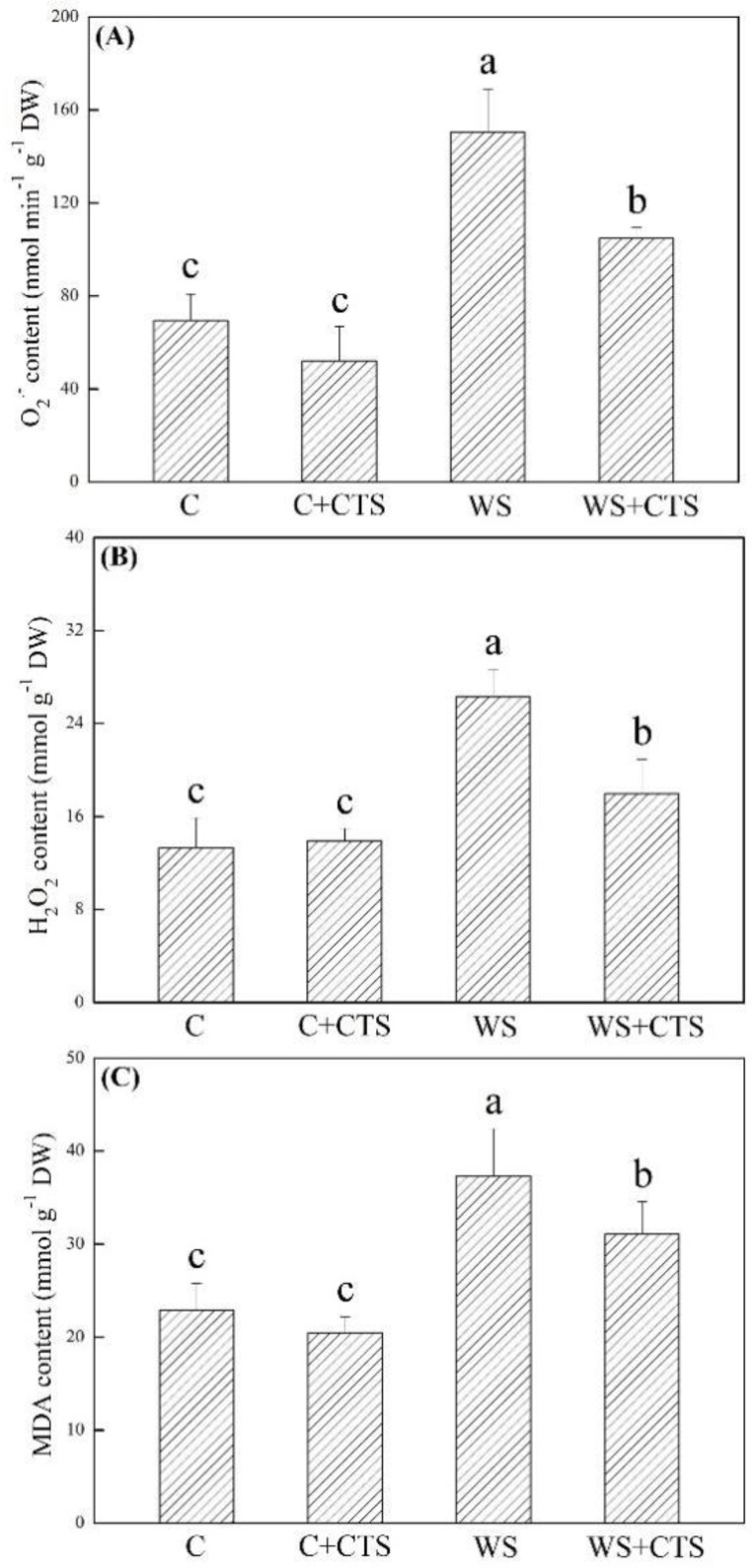
Seeds priming with chitosan affected (**A**) superoxide anion (O_2_^−^) content, (**B**) hydrogen peroxide (H_2_O_2_) content, and (**C**) malondialdehyde (MDA) content in seedlings after 7 days of germination under water stress. Vertical bars above columns indicate ± SE of mean, and different letters above columns indicate significant difference (*p* < 0.05). C, control (seeds pretreated with H_2_O germinated under normal condition); C+CTS, control + CTS (seeds pretreated with CTS germinated under normal condition); WS, water stress (seeds pretreated with H_2_O germinated under water stress condition); WS+CTS, water stress + CTS (seeds pretreated with CTS germinated under water stress condition).

**Figure 5:**
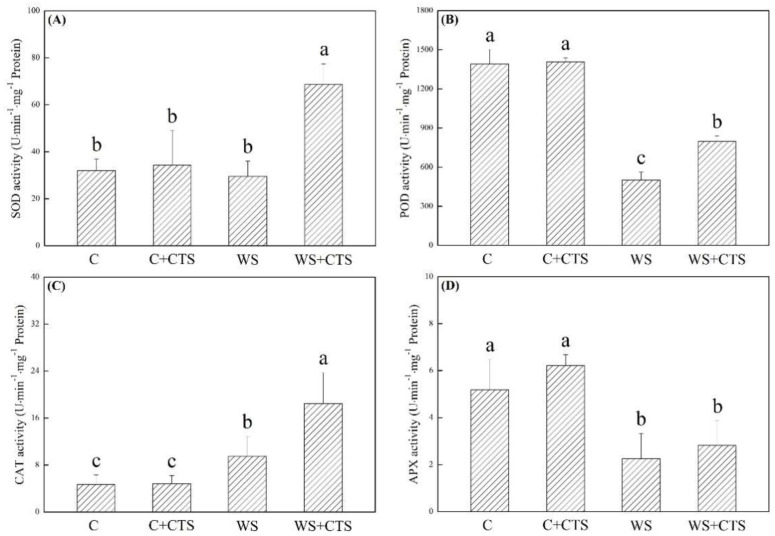
Seeds priming with chitosan affected (**A**) superoxide dismutase (SOD) activity, (**B**) peroxidase (POD) activity, (**C**) catalase (CAT) activity, and (**D**) ascorbate peroxidase (APX) activity in seedlings after 7 days of germination under water stress. Vertical bars above columns indicate ± SE of mean, and different letters above columns indicate significant difference (*p* < 0.05). C, control (seeds pretreated with H_2_O germinated under normal condition); C+CTS, control + CTS (seeds pretreated with CTS germinated under normal condition); WS, water stress (seeds pretreated with H_2_O germinated under water stress condition); WS+CTS, water stress + CTS (seeds pretreated with CTS germinated under water stress condition).

ROS (O_2_^−^ and H_2_O_2_) and MDA significantly accumulated in seedlings after 7 days of germination under water stress (Figure 4A–D). A 30%, 32%, or 16% lower O_2_^.–^, H_2_O_2_, or MDA content was detected in the WS+CTS treatment as compared to the WS treatment under water stress, respectively (Figure 4A–C). As compared to normal condition, SOD activity did not significantly change in the WS treatment but significantly increased in the WS+CTS treatment (Figure 5A). Water stress inhibited the POD activity but improved the CAT activity in both of WS and WS+CTS treatments (Figure 5B,C). Seedlings germinated from seeds priming with the CTS showed significantly higher POD and CAT activities than seedlings without CTS priming (Figure 5B,C). APX activity significantly decreased under water stress, and no significant difference in APX activity was observed between the WS and WS+CTS (Figure 5D).

### 3.3. Seeds Priming with CTS Affected Genes Expression Levels Involved in Dehydration-Responsive Pathway under Water Stress

**Figure 6:**
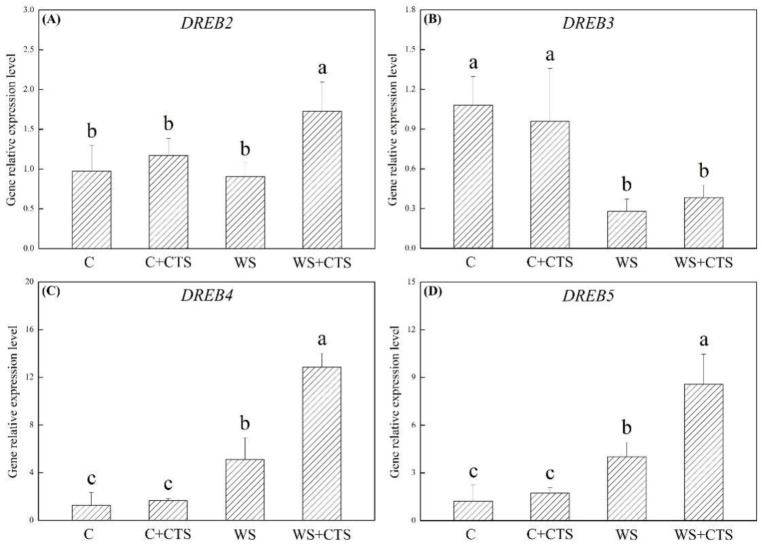
Seeds priming with chitosan affected genes expression levels of (**A**) *DREB2*, (**B**) *DERB3*, (**C**) *DREB4*, and (**D**) *DREB5* encoding different types of dehydration responsive element-binding proteins in seedlings after 7 days of germination under water stress. Vertical bars above columns indicate ± SE of mean, and different letters above columns indicate significant difference (*p* < 0.05). C, control (seeds pretreated with H_2_O germinated under normal condition); C+CTS, control + CTS (seeds pretreated with CTS germinated under normal condition); WS, water stress (seeds pretreated with H_2_O germinated under water stress condition); WS+CTS, water stress + CTS (seeds pretreated with CTS germinated under water stress condition).

**Figure 7:**
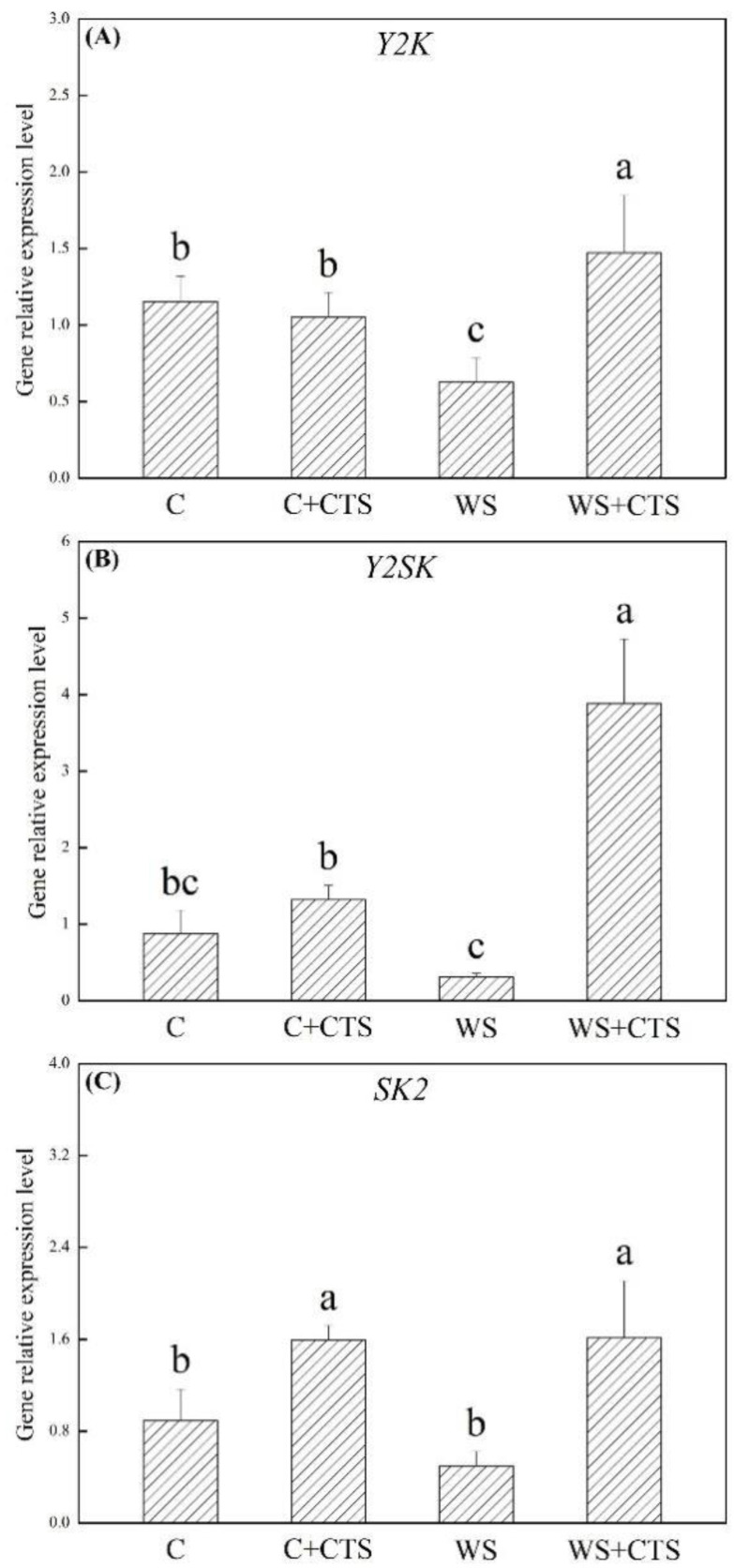
Seeds priming with chitosan affected genes expression levels of (**A**) *Y2K*, (**B**) *Y2SK*, and (**C**) *SK2* encoding different types of dehydrins in seedlings after 7 days of germination under water stress. Vertical bars above columns indicate ± SE of mean, and different letters above columns indicate significant difference (*p* < 0.05). C, control (seeds pretreated with H_2_O germinated under normal condition); C+CTS, control + CTS (seeds pretreated with CTS germinated under normal condition); WS, water stress (seeds pretreated with H_2_O germinated under water stress condition); WS+CTS, water stress + CTS (seeds pretreated with CTS germinated under water stress condition).

Relative expression levels of genes encoding dehydration-responsive element-binding proteins, including *DREB2*, *DREB3*, *DREB4*, and *DREB5,* are shown in Figure 6A–D. *DERB2* expression level was not affected significantly by water stress in the WS treatment, whereas it was significantly increased in the WS+CTS treatment (Figure 6A). *DERB3* expression level was inhibited significantly by water stress in both of the WS and WS+CTS, and there was no significant difference in the *DERB3* expression level between the WS and WS+CTS (Figure 6B). Water stress induced more pronounced increases in the *DREB4* and *DREB5* expression in the WS+CTS than that in the WS (Figure 6C,D). As compared to normal condition, water stress inhibited *Y2K* and *Y2SK* expression levels in the WS but up-regulated the *Y2K* and *Y2SK* expression levels in the WS+CTS (Figure 7A,B). The CTS priming significantly up-regulated the *SK2* expression level in seedling under normal condition and water stress (Figure 7C).

## 4. Discussion

Water stress decreases turf quality and also increases maintenance cost, especially in arid and semi-arid regions worldwide [34]. White clover is a leguminous ground cover plant that is widely used for urban landscaping and conservation of water and soil [35]. As compared to other leguminous species such as alfalfa, white clover is more susceptible to water deficit at the germination stage. Seed priming with bioactive compound has become an important agronomic strategy for improving seed vigor and germination under normal and stress conditions [36]. It has been proven that white clover seed priming with a low concentration of NaCl solution could significantly mitigate adverse effects induced by water stress, including declines in GP, GV, SVI, and radicle length [37]. Recent research also showed that white clover seeds soaking in an appropriate dose of diethyl aminoethyl hexanoate solution before being geminated under water stress effectively improved germination rate, root length, and shoot length of seedlings [23]. In addition, seed coating with CTS has been reported to significantly promote GP and seedling growth of hybrid rice under salt stress [38]. Our study demonstrated that seed priming with the CTS showed better GP, GV, GI, dry weight, root length, and shoot length of seedlings than those seeds primed with H_2_O under water stress. Current findings indicated that the CTS could be used as a beneficial elicitor to improve seed germination under stressful conditions.

Drought-induced high amounts of ROS in cells caused lipid peroxidation, protein degradation, and membrane leakage, resulting in retarded growth, premature senescence, and even death [39]. The overaccumulation of ROS (O_2_^−^ and H_2_O_2_) and the aggravation of membrane lipid peroxidation were observed in our current study when white clover seeds germinated under water-limited conditions. Previous study has found that zinc priming ameliorated adverse effects of drought stress associated with enhancement in total antioxidant capacity and reduction in membrane lipid peroxidation during seed germination [19]. In addition, the regulatory role of CTS in activating the antioxidant defense system to scavenge free radicals has also been reported in response to water stress. For example, the CTS coating could mitigate drought-induced oxidative damage by activating SOD, CAT, and POD activities in wheat seedlings [40]. A combination of seed priming and foliar application of CTS improved shoot and root growth as well as antioxidant enzyme activities, including POD and APX in rice seedling under drought stress [41]. Seeds pretreated by exogenous CTS increased drought tolerance in alfalfa through enhancing the antioxidant defense system [42]. White clover seed priming with CTS significantly alleviated oxidative damage induced by water stress through improving ROS-scavenging enzyme activities, including SOD, POD, and CAT, which indicated the beneficial function of CTS in maintaining ROS homeostasis for better adaptation to a water-deficit environment during early seedling establishment.

The DREBs family is considered one of the most critical classes of TFs in relation to drought tolerance in plants [43]. DREBs regulate stress-defensive genes expression by binding to the DRE/C-repeat core component of these downstream genes under different abiotic stresses [44]. It has been found that transgenic tobacco overexpressing an *RcDREB 5-A* showed better growth and higher biomass than non-transgenic lines in response to drought stress [45]. Similarly, up-regulation of *PcDREB2A* could significantly improve drought tolerance of *Arabidopsis* [46]. On the contrary, RNAi-tomato plants exhibited a significantly lower expression level of *SlDREB2* and higher lipid membrane peroxidation than the wild-type under drought stress [47]. During seed germination, significant increases in expression levels of different types of *DREBs* are also propitious to achieve stress tolerance. For instance, drought tolerance of transgenic *Arabidopsis* overexpressing an *AmDREB2* was improved significantly at the seed-germination stage [48]. A *SgDREB2* overexpression in *Arabidopsis* increased the seed-germination rate, seedlings survival rate, and antioxidant enzyme activities, including SOD and APX, under drought stress, suggesting that *SgDREB2* regulated drought tolerance involved in antioxidant defense [49]. Exogenous CTS priming significantly up-regulated expression levels of *DREB2*, *DREB4*, and *DREB5* during white clover seed germination, which indicated that the potential role of CTS in regulating adaptability to water stress could be associated with the DREB-responsive pathway.

DHNs are diverse classes of stress-responsive proteins that are regulated by the DREBs [50]. DHNs quickly accumulate during seed germination or when plants suffer dehydration due to their positive functions as chaperones, ROS scavengers, and osmoprotectants in cells [51]. Previous study has proven that white clover seed priming with DA-6 significantly mitigated adverse effects of water stress on seed germination and seedling establishment in relation to significant accumulation of DHN and upregulation of *Y2K*, *Y2SK*, and *SK2* genes encoding different types of DHNs [23]. Enhanced *AnDHN* or *CaDHN3* expression in *Arabidopsis* increased seed germination and initial root length under drought stress and also promoted antioxidant capacity to alleviate drought-induced ROS accumulation in seedlings [52,53]. However, silencing of the *CaDHN3* in pepper (*Capsicum annuum* L.) plants significantly decreased drought tolerance, as demonstrated by more accumulation of ROS and MDA contents than the wild-type [53]. A recent study of Decena et al. found that *DHNs* expression among 32 *Brachypodium* grass ecotypes was highly correlated with drought-responsive traits, such as plant biomass and water-use efficiency, and drought-tolerant ecotypes often had higher expression levels of *DHNs* in response to drought stress [54]. Water stress could also induce more or higher *DHNs* expression in drought-tolerant Kentucky bluegrass (*Poa pratensis*) germplasms [55]. Our findings indicated that seed priming with CTS activated the expression of *Y2K-*, *Y2SK-*, and *SK2*-encoding DHNs, which could be a key factor affecting water-stress tolerance in white clover.

## 5. Conclusions

Water stress significantly decreased seed germination characteristics and hindered seedling establishment. Seed priming with CTS could be used as a simple, effective, economical, and environmentally friendly technique to improve germination and seedling establishment under water-deficit conditions. Stress-induced overaccumulation of ROS damaged cell membrane, leading to membrane lipid peroxidation, but this symptom could be significantly alleviated by the CTS priming through activating SOD, POD, and CAT activities. In addition, seeds pretreated with CTS exhibited significantly higher expression levels of *DREB2*, *DREB4*, *DREB5*, *Y2K*, *Y2SK*, and *SK2* than those seeds without the CTS priming. Current findings indicated that the CTS-induced tolerance to water stress could be associated with the enhancement in dehydration-responsive pathway during seed germination.

## Figures and Tables

**Table 1:** Primer sequences and GeneBank accession numbers of genes.

Target Gene	Accession No.	Forward Primer (5′-3′)	Reverse Primer (5′-3′)	Tm (°C)
SK2	GU443960.1	TGGAACAGGAGTAACAACAGGTGGA	TGCCAGTTGAGAAAGTTGAGGTTGT	58
Y2K	JF748410.1	AGCCACGCAACAAGGTTCTAA	TTGAGGATACGGGATGGGTG	60
Y2SK	GU443965.1	GTGCGATGGAGATGCTGTTTG	CCTAATCCAACTTCAGGTTCAGC	60
DREB2	EU846194.1	CAAGAACAAGATGATGATGGTGAAC	AAGAAGAAGAATTGGAGGAGTCATG	58
DREB3	EU846196.1	GCTCAATAGGACTCAACCAACTCAC	TGACGTTGTCTAACTCCACGGTAA	58
DREB4	EU846198.1	CTTGGTTGTGGAGATAATGGAGC	AAGTTGCAATCTGAATTCTGAGGAC	58
DREB5	EU846200.1	GCGATAGGTTCAAAGAAAGGGTG	AGAGCAGCATCTTGAGCAGTAGG	58
β-Actin	JF968419	TTACAATGAATTGCGTGTTG	AGAGGACAGCCTGAATGG	58

## Data Availability

All data presented in this study are available in the article.
